# Comprehensive Genetic Screening of *KCNQ4* in a Large Autosomal Dominant Nonsyndromic Hearing Loss Cohort: Genotype-Phenotype Correlations and a Founder Mutation

**DOI:** 10.1371/journal.pone.0063231

**Published:** 2013-05-23

**Authors:** Takehiko Naito, Shin-ya Nishio, Yoh-ichiro Iwasa, Takuya Yano, Kozo Kumakawa, Satoko Abe, Kotaro Ishikawa, Hiromi Kojima, Atsushi Namba, Chie Oshikawa, Shin-ichi Usami

**Affiliations:** 1 Department of Otorhinolaryngology, Shinshu University School of Medicine, Matsumoto, Japan; 2 Department of Otolaryngology, Toranomon Hospital, Tokyo, Japan; 3 Department of Otorhinolaryngology, Jichi Medical University, Tochigi, Japan; 4 Department of Otorhinolaryngology, Jikei University School of Medicine, Tokyo, Japan; 5 Department of Otorhinolaryngology, Hirosaki University School of Medicine, Hirosaki, Japan; 6 Department of Otorhinolaryngology, Kyushu University School of Medicine, Fukuoka, Japan; Odense University hospital, Denmark

## Abstract

The present study of *KCNQ4* mutations was carried out to 1) determine the prevalence by unbiased population-based genetic screening, 2) clarify the mutation spectrum and genotype/phenotype correlations, and 3) summarize clinical characteristics. In addition, a review of the reported mutations was performed for better understanding of this deafness gene. The screening using 287 probands from unbiased Japanese autosomal dominant nonsyndromic hearing loss (ADNSHL) families identified 19 families with 7 different disease causing mutations, indicating that the frequency is 6.62% (19/287). While the majority were private mutations, one particular recurrent mutation, c.211delC, was observed in 13 unrelated families. Haplotype analysis in the vicinity of c.211delC suggests existence of a common ancestor. The majority of the patients showed all frequency, but high-frequency predominant, sensorineural hearing loss. The present study adds a new typical audiogram configuration characterized by mid-frequency predominant hearing loss caused by the p.V230E mutation. A variant at the N-terminal site (c. 211delC) showed typical ski-slope type audiogram configuration. Concerning clinical features, onset age was from 3 to 40 years old, and mostly in the teens, and hearing loss was gradually progressive. Progressive nature is a common feature of patients with *KCNQ4* mutations regardless of the mutation type. In conclusion, *KCNQ4* mutations are frequent among ADNSHL patients, and therefore screening of the gene and molecular confirmation of these mutations have become important in the diagnosis of these conditions.

## Introduction

Autosomal dominant nonsyndromic hearing loss (ADNSHL) is extremely heterogeneous. To date, more than 60 DFNA loci have been identified and 27 genes for DFNA have been identified (Van Camp G, Smith RJH. Hereditary Hearing Loss Homepage: http://hereditaryhearingloss.org). Genetic testing has become crucial for precise diagnosis, progression estimation, and selection of ideal intervention. However, due to such genetic heterogeneity and lack of recurrent mutations, routine genetic testing for ADNSHL has lagged. Linkage analysis is a powerful tool to identify a responsible gene for ADNSHL, but in the usual clinical setting, only a limited number of samples are available and this is insufficient for linkage analysis. Among ADNSHL genes, several are frequent, for example, *WFS1, KCNQ4, COCH, GJB2, MYO1A*, and *TECTA* [1]. Based on the number of reported mutations, the *KCNQ4* gene (responsible gene for DFNA2) is known to be one of the most frequent responsible genes for ADNSHL [1]. *KCNQ4*, a member of the voltage-gated potassium channel family, plays a role in potassium recycling in the inner ear [2]. In this 695-amino acid protein there are six transmembrane domains and a hydrophobic P-loop region, which is between the transmembrane domains S5 and S6 (residues 259 to 296). A channel pore, containing a potassium ion-selective filter, is formed by the P-loop domain. Channel function of this selectivity filter is eliminated by pore region mutations [2]. DFNA2-associated hearing loss has been reported to be typically late onset high frequency-involved and progressive over time, as opposed to early onset and severe loss in recessive forms [3]. To date, more than ten pathologic mutations have been identified in *KCNQ4* and they are mostly missense mutations with a dominant-negative mechanism [3]. It was a matter of interest to know the prevalence of *KCNQ4* mutations to be found through unbiased population-based genetic screening. In this study, we performed the screening in a comprehensive manner to establish the mutation spectrum and genotype/phenotype correlations associated with this type of ADNSHL. Also, we were interested to know whether there are any recurrent mutations. In addition, we reviewed the reported mutations for better understanding of this deafness gene. We found that *KCNQ4* is frequent among ADNSHL patients, and therefore an important causative gene to be screened.

## Materials and Methods

### Subjects and clinical evaluation

The subjects participating in this study were 287 probands, each from an independent Japanese ADNSHL family. Whether or not progression was present was based on anamnestic evaluation. None of the subjects had any other associated neurological signs, visual dysfunction or diabetes mellitus. The control group was 252 unrelated Japanese individuals with normal hearing evaluated by auditory testing. The average threshold in the conversation frequencies (0.5 kHz, 1 kHz, 2 kHz) was calculated for the better ear, and severity of hearing loss was noted to be normal (−19 dB) in 24 subjects, mild (20–39 dB) in 69 subjects, moderate (40–69 dB) in 132 subjects, severe (70–94 dB) in 23 subjects, and profound (≥95 dB) in 24 subjects. Subjects with high frequency hearing loss only at 4 kHz and 8 kHz were classified as normal because they had normal hearing at 0.5, 1 and 2 kHz. Hearing loss severity was not obtained for 15 subjects. All probands' pure-tone thresholds were recorded on the frequencies of 125, 250, 500, 1000, 2000, 4000, and 8000 Hz.

### Ethics Statement

All subjects or next of kin, caretakers, or guardians on the behalf of the minors/children gave prior written informed consent for participation in the project, and the Ethical Committee of Shinshu University approved the study and the consent procedure.

### Mutation analysis

All fourteen exons and flanking intronic sequences of the *KCNQ4* gene were amplified by polymerase chain reaction PCR. Primers were designed to flank all of the exon-intron boundaries through use of the Primer3 web based server. Each genomic DNA sample (40 ng) was amplified using Multiplex PCR Assay Kit (Takara, Shiga, Japan) for 5 min at 95°C, followed by 40 three-step cycles of 94°C for 30 s, 60–67.6°C for 90 s, and 72°C for 90 s, with a final extension at 72°C for 10 min, ending with a holding period at 4°C in a Perkin-Elmer thermal cycler. The PCR products varied in size at about 100–400 bp, and they were treated with 0.1 ul exonuclease I (Amersham) and 1 ul shrimp alkaline phosphatase (Amersham) and by incubation at 37°C for 30 min, and inactivation at 80°C for 15 min. After the products were purified, we performed standard cycle sequencing reaction with ABI Big Dye terminators in an ABI 3100 autosequencer (Applied Biosystems).

Computer analysis to predict the effect of missense variants on the protein function was performed with wANNOVAR (http://wannovar.usc.edu) including the functional prediction software listed below. PhyloP (http://hgdownload.cse.ucsc.edu/goldenPath/hg18/phyloP44way/), Sorting Intolerant from Tolerant (SIFT; http://sift.jcvi.org/), Polymorphism Phenotyping (PolyPhen2; http://genetics.bwh.harvard.edu/pph2/), LRT (http://www.genetics.wustl.edu/jflab/lrt_query.html), and MutationTaster (http://www.mutationtaster.org/).

### Haplotype analysis

Haplotype pattern within the 1Mbp region surrounding position c.211, where the frequent Japanese mutation c.211delC was found, was analyzed using a set of 48 single nucleotide polymorphisms (SNPs) (21 sites upstream and 27 sites downstream). Haplotype analysis was performed by the direct sequencing method described above.

### Statistical analysis of progression of hearing loss

Each subject's ages at the time of examination and their pure tone thresholds were plotted for detailed progression analysis with 125, 250, 500, 1000, 2000, 4000, 8000 Hz, respectively. The average progressive rates of hearing loss (db/year) were calculated by linear regression lines, and analysis of difference of the rates was performed using analysis of covariance (ANCOVA) with SPSS ver19 software.

## Results

### Mutation analysis

**Figure 1:**
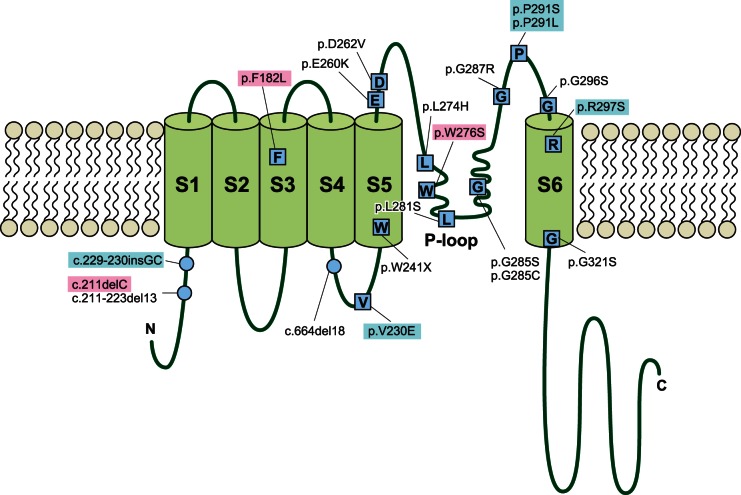
Localization of 20 *KCNQ4* mutations reported in previous studies in the protein. The 6 transmembrane domains (S1–S6) and the P-loop, located between S5 and S6, are shown. 5 mutations are concentrated in a narrow P-loop range. Mutations with pink and blue shadows; possible mutations detected in this study. Blue indicates novel mutations. Original schema is modified from Mencía A (2008) [14].

Direct DNA sequencing identified 8 possible disease-causing mutations among 20 autosomal dominant families (Table 1). There were one deletion mutation (c.211delC), one insertion mutation (c.229_230*ins*GC), and 6 missense mutations (p.F182L, p.V230E, p.W276S, p.P291S, p.P291L, p.R297S) (Table 1). These included 5 novel and three previously reported pathologic mutations: c.211delC, p.F182L, and p.W276S (Table 1, Fig. 1). However, we excluded p.F182L as it is unlikely to be pathologic, according to the prediction program (Table 1). p.F182L was also found in a control sample with normal audiogram (Table 1). Therefore, 7 pathologic mutations from 19 families were found in a total of 287 ADNSHL families in this study (Fig. S1). Concerning the domains in which the 7 mutations were localized, 2 mutations were found in the N-terminal cytoplasmic domain, one mutation in the S4–S5 linker domain, 3 mutations in the pore region and the P-loop region, and one mutation in the S-6 transmembrane domain (Table 1, Fig. 1).

**Table 1:** *KCNQ4* mutations found in this study together with previously reported mutations.

Functional Prediction													
Nucleotide Change	Amino Acid Change	Exon	Position	Alleles in Control Chr	SIFT	P2 D.S.	PhyloP	LRT	Mut Taster	GERP++	Study location	No of Fm	Reference
c.211_223del13	p. Q71fs	1	N-term cyto	?	–	–	–	–	–	–	Belgium	1	Coucke, et al. (1999)
c.211delC	p. Q71fs	1	N-term cyto	0/252	–	–	–	–	–	–	Japan	14	Kamada, et al. (2006), This report
* c.229_230insGC	**p.H77fs**	**1**	**N-term cyto**	**0/252**	–	–	–	–	–	–	**Japan**	**1**	**This report**
c.546C>G	p.F182L	4	S3 trans	0/100, 1/252	T (0.00)	B (0.01)	C (0.97)	N (0.999853)	D (0.88)	3.43	Taiwan, Japan	3	Su, et al. (2007), This report
c.664_681del18	p.G215_220del6	4	S4-S5 linker	0/100	–	–	–	–	–	–	Korea	1	Baek, et al. (2010)
* c.689T>A	**p.V230E**	**4**	**S4–S5 linker**	**0/252**	**D (1.00)**	**D (0.97)**	**C (0.99)**	**D (0.999999)**	**D (0.99)**	**4.61**	**Japan**	**1**	**This report**
c.725G>A	p.W241X	5	S5 trans	0/100	–	–	–	–	–	–	USA	1	Hildebrand, et al. (2008)
c.778G>A	p.E260K	5	S5 trans	0/100	D (1.00)	D (0.99)	C (0.99)	D (1.00)	D (0.99)	4.73	USA	1	Hildebrand, et al. (2008)
c.785A>T	p.D262V	5	S5 trans	0/100	D (1.00)	D (0.99)	C (0.99)	D (1.00)	D (0.99)	4.73	USA	1	Hildebrand, et al. (2008)
c.821T>A	p.L274H	5	PR (P)	?	D (1.00)	D (0.99)	C (0.99)	D (1.00)	D (1.00)	4.73	Neth	2	Van Hauwe, et al. (2000), De Heer, et al. (2011)
c.827G>C	p.W276S	5	PR (P)	0/252	D (1.00)	D (1.00)	C (0.99)	D (1.00)	D (1.00)	4.73	Neth, Japan	4	Coucke, et al. (1999), Akita et al. (2001), Van Camp, et al. (2002), Topsakal, et al. (2005)
c.842T>C	p.L281S	6	PR (P)	0/96	D (1.00)	Pr (0.84)	C (0.99)	D (1.00)	D (1.00)	5.14	USA	1	Talebizadeh, et al. (1999)
c.853G>T	p.G285C	6	PR (P)	?	D (1.00)	D (1.00)	C (0.99)	D (0.999999)	D (1.00)	5.14	USA	1	Coucke, et al. (1999)
c.853G>A	p.G285S	6	PR (P)	0/150	D (1.00)	D (0.99)	C (0.99)	D (0.999999)	D (1.00)	5.14	France	1	Kubisch, et al. (1999)
c.859G>C	p.G287R	6	PR (P)	0/274	D (1.00)	D (0.99)	C (0.99)	D (1.00)	D (1.00)	5.14	USA	1	Arnett, et al. (2011)
* c.871C>T	**p.P291S**	**6**	**PR (P)**	**0/252**	**D (1.00)**	**D (1.00)**	**C (0.99)**	**D (1.00)**	**D (1.00)**	**5.14**	**Japan**	**1**	**This report**
* c.872C>T	**p.P291L**	**6**	**PR (P)**	**0/252**	**D (1.00)**	**D (1.00)**	**C (0.99)**	**D (1.00)**	**D (1.00)**	**5.14**	**Japan**	**1**	**This report**
c.886G>A	p.G296S	6	PR	0/100	D (0.99)	D (0.97)	C (0.99)	D (1.00)	D (0.99)	5.14	Spain	1	Mencia, et al. (2008)
* c.891G>T	**p.R297S**	**6**	**S6 trans**	**0/252**	**D (1.00)**	**D (0.99)**	**C (0.99)**	**D (1.00)**	**D (0.95)**	**3.89**	**Japan**	**1**	**This report**
c.961G>A	p.G321S	7	S6 trans	?	D (0.99)	Po (0.31)	C (0.99)	D (1.00)	D (0.99)	4.92	Neth	1	Coucke, et al. (1999)

SIFT, Polyphen-2, PhyloP, LRT, Mutation Taster, and GERP++ are functional prediction scores in which increasing values indicate a probable mutation.

Abbreviations: Chr, chromosomes; P2, PolyPhen2; MutTaser, Mutation Taser; Fm, family; cyto, cytoplasmic; trans, transmembrane; PR, Pore region; (P), P-loop; T, tolerated; D, damaging or deleterious; B, benign; Pr, probably damaging; Po, possibly damaging; C, conserved; N, neutral. Neth, Netherlands; *, Novel mutations found in this study.

### Frequency of KCNQ4 mutations

The frequency of *KCNQ4* mutations found in ADNSHL families in this study was 6.62% (19/287). The most prevalent mutation was c.211delC, at 4.53% (13/287) and it accounted for 68.4% (13/19) of all KCNQ4 mutations.

### Haplotype analysis

**Figure 2:**
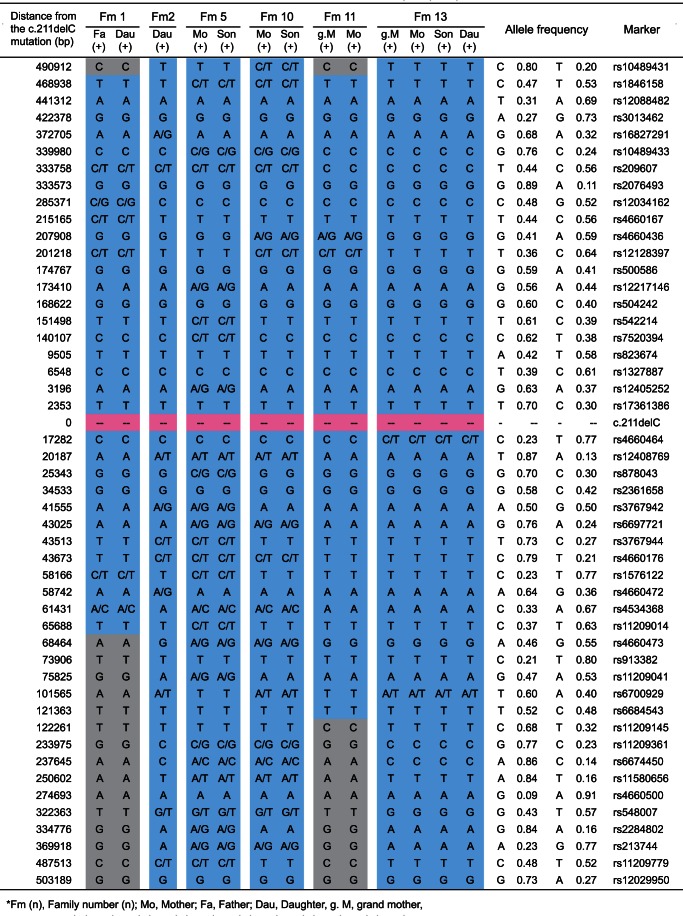
The haplotypes around c.211delC mutation of six families constructed using SNPs are shown. Each column shows an affected allele. Each base is defined by pure segregation analysis in the family. Allele frequencies of SNPs are derived from HapMap JPT+CHB samples. Families 2, 5, 10, and 13 shared a large common region of about more than 1 Mb in their haplotypes (blue). Abbreviation: Fm, Family.

Haplotype pattern within the 1Mbp region surrounding the position of the most frequent mutation c.211delC, was characterized using a set of 48 single nucleotide polymorphisms (SNPs) (21 sites upstream and 27 sites downstream). All patients from 6 families with c.211delC showed an exactly identical pattern in the allele with c.211delC, though the other allele showed a variety of haplotype patterns (Fig. 2).

### Clinical characteristics

Table 2 summarizes clinical characteristics of 36 patients from 19 families with hearing loss caused by the *KCNQ4* mutations, including age at their first visit to the ENT clinic, onset age (age of awareness), audiogram configuration, progression of hearing loss, tinnitus, and vestibular symptoms. The ages at first clinic visits were from 0 to 78 years. Ages of onset (awareness age) ranged from 3 to 40 years old, though the majority became aware when in their teens or younger. Most patients had associated tinnitus, but no vestibular symptoms except in a few cases.

**Table 2:** Clinical features of affected family members associated with KCNQ4 mutations found in this study.

Amino Acid Change	Family – Patient No.	HL onset age (years)	Age at the first visit (years)	Audiogram frequencies	Progression	Tinnitus	Vertigo
Q71fs	1–1	40	48	Ski slope	N/A	N/A	N/A
	1–2	15	15	Ski slope	+	−	−
	2–1	30	47	Ski slope	+	+	−
	3–1	N/A	31	Ski slope	N/A	−	−
	4–1	12	37	Ski slope	+	+	−
	5–1	32	42	Ski slope	−	+	−
	5–2	10	15	Ski slope	+	+	−
	6–1	14	40	Ski slope	+	+	−
	7–1	11	35	Ski slope	+	+	−
	8–1	18	25	Ski slope	+	+	−
	9–1	18	29	Ski slope	+	+	−
	10–1	17	22	Ski slope	+	+	−
	10–2	20	52	Ski slope	+	+	−
	11–1	40	43	Ski slope	+	−	−
	11–2	N/A	73	Ski slope	N/A	−	−
	12–1	22	38	Ski slope	+	+	−
	13–1	35	55	Ski slope	+	+	−
	13–2	25	33	Ski slope	+	+	+
	13–3	11	14	Ski slope	N/A	+	+
	13–4	−	6	Normal (*)	N/A	N/A	N/A
**H77fs**	**14**	**22**	**27**	**Ski slope**	**+**	**+**	−
**V230E**	**15–1**	**40**	**78**	**mid freq**	**+**	**+**	−
	**15–2**	**12**	**39**	**mid freq**	**+**	−	−
	**15–3**	**5**	**5**	**mid freq**	**+**	−	−
	**15–4**	**3**	**3**	**mid freq**	**N/A**	**N/A**	**N/A**
	**15–5**	**N/A**	**0**	**mid freq**	**N/A**	**N/A**	**N/A**
W276S	16–1	8	65	high freq	+	−	+
	16–2	12	46	high freq	+	−	−
	16–3	7	42	high freq	+	−	−
	16–4	8	8	high freq	+	−	+
	16–5	8	6	high freq	+	−	−
**P291S**	**17–1**	**20**	**33**	**high freq**	**+**	**N/A**	**N/A**
**P291L**	**18–1**	**17**	**40**	**high freq**	**N/A**	**N/A**	**N/A**
	**18–2**	**17**	**15**	**high freq**	**N/A**	**N/A**	**N/A**
**R297S**	**19–1**	**39**	**39**	**high freq**	−	**+**	−
	**19–2**	**5**	**5**	**high freq**	**+**	−	−

Abbreviations: HL, hearing loss; mid, middle; freq, frequency; N/A, not applicable.

(*) Six-year-old boy's hearing is normal in spite of having the mutation.

### Genotype/phenotype correlations

**Figure 3:**
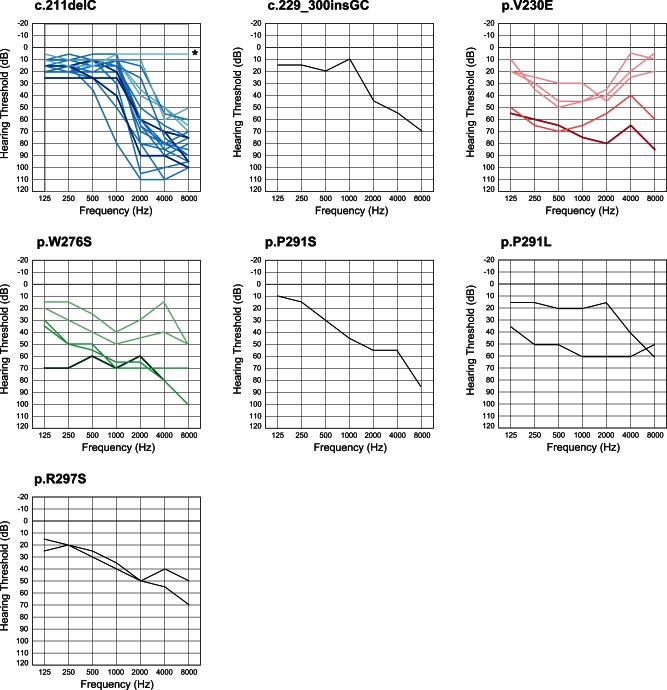
Overlapping audiograms from the better ear for each genotype. In cases of W276S, c.211delC, or V230E, light colored audiograms (green, blue, red) were from individuals aged 19 and under. Dark colored audiograms (green, blue, red) were from the patients aged 20–49 years old, and deep colored audiograms (green, blue, red) are from the patients in their 50 s and over. In family #13 with c.211delC, (*) a six-year-old boy's hearing is normal in spite of having the mutation.

Concerning type of hearing loss, there were some correlations between genotype and phenotype (audiogram configuration). A variant at the N-terminal site (c. 211delC) showed ski-slope type configuration of audiogram with usually nearly normal hearing at 125–1000 Hz. We found this mutation in 20 patients from 13 families and their overlapped audiogram confirmed a similar configuration (Fig. 3). Onset age was from 10 to 40 years old, with most being in theirs teens and hearing loss was gradually progressive with age (Fig. 3, Table 2). The patients who had a variant in the P-loop region (W276S, P291L, P291S) also had high frequency involved hearing loss, but with some deterioration in the lower frequencies as well (Fig. 3). Most of the patients had earlier onset compared to the former phenotype and a progressive nature (Fig. 3, Table 2). The third audiogram configuration was mid-frequency involved hearing loss found in a family with a variant in the S4–S5 linker region (V230E) (Fig. 3). In most family members, onset was before age ten and gradually progressive (Fig. 3, Table 2). Overlapped audiograms were made for three mutations (W276S, c.211delC, V230E) for which there was a large enough number of patients to be analyzed (Fig. 3).

### Therapeutic intervention

Sufficient amplification of hearing aids was obtained in all patients, and no patients received cochlear implantation. An affected subject with W276S (Family-Patient No. 16–2 in Table 2) had used a hearing aid from age 29. Similarly, affected subjects with P291L (Family-Patient No. 18–1) and V230E (Family-Patient No. 15–2) had used hearing aids. None of the affected subjects with c.211delC had a history of hearing aid usage.

### Progression analysis

**Figure 4:**
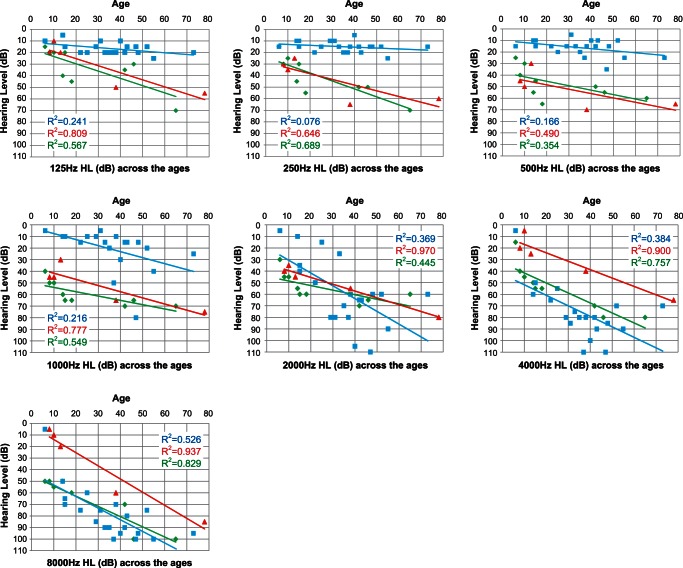
Detailed progression analysis in each frequency. A single audiogram (the better ear) from a single patient was plotted. Gradual progression is characterized regardless of frequency. Average progressive rates of hearing loss (db/year) for the patients with c.211delC, for 125 (0.15) and 250 Hz (0.078) were significantly stable compared to the other two mutations (ANCOVA: p<0.05) and they had milder hearing loss at 500 and 1 KHz (ANCOVA: p<0.05). In contrast, at 4 KHz and 8 KHz, patients with V230E mutations had milder hearing loss compared to the other two mutations (ANCOVA: p<0.05). Each color (green, blue, red) indicates W276S, c.211delC, or V230E, respectively.

Detailed progression analysis in each frequency showed each affected member's age and their pure tone thresholds for 125, 250, 500, 1000, 2000, 4000, 8000 Hz, respectively (Fig. 4). Linear regression lines calculated by the plots are shown in the graph. Regarding the average progressive rates of hearing loss (db/year) for the patients with c.211delC, 125 (0.15) and 250 Hz (0.078) were shown to be significantly stable compared to the other two mutations (ANCOVA: p<0.05). They exhibited milder hearing loss at 500 and 1 KHz (ANCOVA: p<0.05). In contrast, at 4 KHz and 8 KHz, the patients with V230E mutations showed milder hearing loss compared to the other two mutations (ANCOVA: p<0.05).

## Discussion

In this study, we have conducted a comprehensive genetic screening of *KCNQ4* using a large cohort of Japanese ADNSHL patients to establish the mutation spectrum. The *KCNQ4* mutations found in this study together with previously reported mutations (summarized in Table 1) represent an up-dated mutation spectrum for this gene. For missense mutations, we have gone through all reported missense mutations by computer analysis programs, SIFT and PolyPhen2, to predict the effect of missense variants on *KCNQ4* protein function. A missense mutation (p.F182L) was found in one control patient with normal audiogram and the results showed that it is not likely to be a pathologic mutation.

The present study identified 7 possible disease-causing mutations, including 5 novel mutations, in 19 autosomal dominant families. Based on our unbiased population-based genetic screening, the frequency is 6.62% (19/287) of the overall ADNSHL population. These data indicated that *KCNQ4* is one of the important causative genes among ADNSHL patients, particularly in patients with high frequency-involved hearing loss. This frequency is higher than our recently reported frequency (4/139: 2.9%) of *TECTA* in Japanese ADNSHL families [4], therefore *KCNQ4* is found to be currently the most prevalent gene responsible for Japanese ADNSHL patients, and should be the first in line to be analyzed for ADNSHL patients.

Mutations lie in various domains of the *KCNQ4* protein. While the majority are private mutations, one particular recurrent mutation, c.211delC, was observed in 13 unrelated families. In this gene, we have reported that there is a hot spot mutation, p.W276S, in Belgian, Dutch, and Japanese families [5]. Based on haplotype analysis, in the case for c.211delC, it is not likely a hot spot but rather is suggested to be due to a common ancestor. Such recurrent mutations are common in recessive genes such as 235delC, 35delG, 167delT in *GJB2* [6] [7], H723R in *SLC26A4* [8], and P204L in *CDH23* [9]. They are rare in dominant genes, though a mutation in *DFNA5* that causes autosomal dominant sensorineural hearing loss was reported to arise from a common ancestor [10]. Together with specific audiogram configuration, this may facilitate genetic testing for ADNSHL with a particular phenotype.

Table 2 summarizes clinical characteristics including hearing threshold, severity, onset age (age of awareness), progressiveness of hearing loss, and vestibular symptoms. Age of onset (awareness of hearing loss) ranged from 3 to 40 years old, though the majority of the patients were in their first decade of life. Many of the mutations were accumulated in the P-loop region as described before [3] [11] [12], but mutations were also found in the other domains (Table 1, Fig. 1). There were some correlations between genotype and phenotype (Fig. 3). Overlapped audiograms showed characteristic high frequency involved hearing loss in the majority of the patients with *KCNQ4* mutations. Unique audiograms were shown in the patients with c.211delC and p.V230E. The patients associated with c.211delC showed so-called ski slope hearing loss (high frequency involved hearing loss with nearly normal hearing at lower frequencies). Patients with p.V230E showed mid-frequency involved hearing loss.

It has been known that DFNA2 shows high-frequency involved hearing loss [3] [13] [14]. Based on collected audiograms from the patients with *KCNQ4*, an effective selection algorithm named “Audioprofile” has been proposed and many mutations have actually been successfully identified [13]. The present large cohort study allowed us to confirm and extend the genotype-phenotype correlations. It added a new type of audiogram configuration characterized by mid-frequency predominant hearing loss caused by a *KCNQ4* mutation (Fig. 3). Family #15 had a heterozygous T>A transition at nucleotide 689 in exon 4, which results in a Val to Glu substitution (V230E). This mutation was present in all five affected individuals, and not present in two unaffected family members. None of the 252 normal controls had this mutation. Prediction programs indicated that this mutation is likely to be pathologic. So far mid-frequency predominant hearing loss has been reported with *TECTA* mutations [4]. In this family, we sequenced for *TECTA* to find a mutation, but none were found (data not shown). A different *KCNQ4* mutation (c.664_681del) within the same domain as this mutation was reported to cause high-frequency involved hearing loss, suggesting that the phenotype is not domain-specific [15]. The V230E mutation is a missense mutation that substitutes a nonpolar and aliphatic valine for a negatively charged glutamate. This single base substitution is located adjacent to the S4 transmembrane domain that has a key role as a voltage sensor. The V230E mutation may therefore change sensitivity of voltage sensor and have an affect on passage of potassium through the cell membrane.

The ski-slope type audiogram configuration found in the patients with c.211delC is also a striking characteristic phenotype (Fig. 3). Single families associated with c.211delC [16] and c.211_223del13 [17] have previously been reported to show ski-slope audiograms. The audiogram collection in this study further generalized this phenotype in the N-terminal site.

Analysis of the different frequencies found evident quickly progressive hearing loss in the middle frequencies, therefore those patients may be at risk for rapid deterioration of speech understanding during the time course. Patients with ski-slope type audiograms sometimes have difficultly in being fitted with hearing aids, but Electric Acoustic Stimulation (EAS) has recently been shown to be effective for those patients with high frequency involved hearing loss [18]. The present data on progression speed showed more stable hearing at low frequencies (125 and 250Hz) (Fig. 4), indicating EAS will be the potential therapeutic intervention for the patients with this particular mutation.

Progressive nature is a common feature of the patients with *KCNQ4* mutations regardless of the particular mutation (Fig. 3). Overlapped audiograms of all subjects with W276S, c.211delC, or V230E mutations showed the progressive nature of hearing loss regardless of the mutation type. However, no patients received cochlear implants in this cohort, suggesting that profound hearing loss may seldom be seen though their hearing loss has a progressive nature.

In conclusion, *KCNQ4* is frequent among ADNSHL patients, and therefore screening for this gene and molecular confirmation of *KCNQ4* mutations have become important in the diagnosis of these conditions.

## Supporting Information

Figure S1 Pedigrees of the *KCNQ4* mutation families and detected mutations.
(PDF)
