# Supplementary figures and images for: Comprehensive Genetic Screening of KCNQ4 in a Large Autosomal Dominant Nonsyndromic Hearing Loss Cohort: Genotype-Phenotype Correlations and a Founder Mutation
Source: PLoS One. 2013 May 23;8(5):e63231. doi: 10.1371/journal.pone.0063231 (PMC3662675; doi:10.1371/journal.pone.0063231)

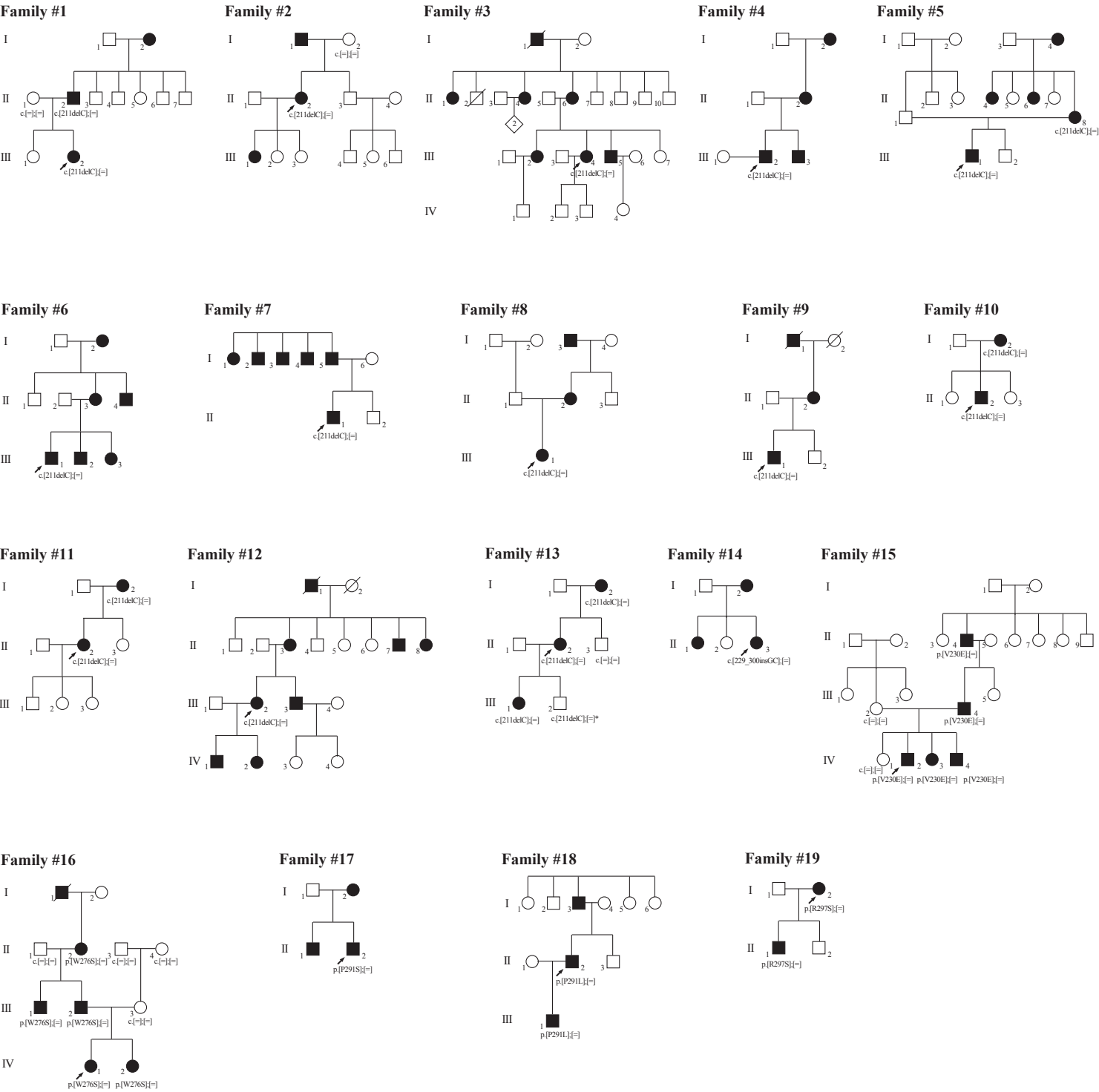

Supplement: Figure S1 — Pedigrees of the KCNQ4 mutation families and detected mutations. (PDF) [file pone.0063231.s001.pdf]
